# Work–family conflict and occupational fatigue among clinical nurses in China: a latent profile analysis

**DOI:** 10.3389/fpubh.2026.1848429

**Published:** 2026-06-18

**Authors:** Zhi Zeng, Yazhi He, Xiang Liao, Sumei Zhou, Guoqin Mao

**Affiliations:** 1 Endoscopy Center, Deyang People's Hospital, Deyang, Sichuan, China; 2 Department of Neurosurgery, Deyang People's Hospital, Deyang, Sichuan, China; 3 Department of Thoracic Surgery II, Sichuan Cancer Hospital and Institute, Sichuan Cancer Center, School of Medicine, University of Electronic Science and Technology of China, Chengdu, Sichuan, China

**Keywords:** clinical nurses, conservation of resources theory, latent profile analysis, occupational fatigue, work–family conflict

## Abstract

**Objective:**

To examine the impact of work–family conflict (WFC) on occupational fatigue among clinical nurses in China and to identify the heterogeneity of occupational fatigue using latent profile analysis (LPA).

**Methods:**

A multicenter cross-sectional survey was conducted between August and September 2025, involving 332 clinical nurses from six tertiary hospitals in Sichuan Province, China. Data were collected using a general information questionnaire, the Fatigue Assessment Instrument (FAI), and the Work–Family Conflict Scale (WFC). Latent profile analysis was applied to identify distinct subgroups of occupational fatigue. Univariate analyses and multinomial logistic regression were performed to explore factors associated with different fatigue profiles.

**Results:**

A total of 332 valid questionnaires were included (response rate: 97.08%). The mean occupational fatigue score was 22.07 ± 2.59. Three latent profiles were identified: the low-fatigue group (8.13%), moderate-fatigue group (31.02%), and high-fatigue group (60.84%). Significant differences were observed across the three groups in daily working hours, weekly working days, and levels of work–family conflict (*P* < 0.05). Notably, work–family conflict increased progressively with fatigue severity. Multinomial logistic regression analysis showed that work–family conflict, daily working hours, and weekly working days were significantly associated with membership in both the moderate-fatigue group and high-fatigue group (*P* < 0.05).

**Conclusions:**

Occupational fatigue among clinical nurses exhibits significant heterogeneity and can be classified into three latent profiles. Work–family conflict was significantly associated with occupational fatigue profiles among clinical nurses. Nursing managers may adopt a profile-based approach to identify fatigue patterns and implement targeted interventions from an individual-differences perspective to reduce occupational fatigue among nurses.

## 1 Introduction

Nurses play a pivotal role in maintaining continuity of care and ensuring patient safety within healthcare systems (1). With the growing global demand for healthcare services and the increasing complexity of nursing tasks, shortages in the nursing workforce have become a widespread challenge, placing nurses under sustained high workloads and responsibility (2, 3). According to the World Health Organization, there remains a global shortage of approximately 5.9 million nurses, and more than 80% of nurses experience varying degrees of occupational fatigue, indicating a substantial burden of work-related strain in this population (4, 5).

In China, despite the total number of registered nurses reaching 5.63 million by the end of 2023, the nurse-to-population ratio remains relatively low (approximately 3.7 per 1,000 population), and the uneven distribution of nursing resources persists (6, 7). This imbalance between high work demands and insufficient recovery opportunities leads to sustained depletion of physical and psychological resources, resulting in the accumulation of fatigue. Consequently, occupational fatigue has become a prevalent and pressing public health concern among nurses (8).

Occupational fatigue is defined as a multidimensional state of functional impairment caused by prolonged exposure to work demands combined with insufficient recovery, characterized by increased exhaustion, reduced alertness, and diminished recovery capacity (9). Among nurses, occupational fatigue not only compromises clinical judgment and work performance but is also associated with adverse outcomes such as nursing errors, impaired cognitive performance, patient safety risks, and increased turnover intention (10, 11). Previous studies have further demonstrated that fatigue may negatively affect nurses' attention, decision-making ability, and overall patient safety outcomes (10, 11). In addition, prolonged working hours, shift work, and excessive workload have been identified as important contributors to occupational fatigue and impaired recovery among nurses (12).

From a theoretical perspective, the Conservation of Resources (COR) theory posits that individuals strive to obtain, retain, and protect their resources, and fatigue arises when resource demands exceed resource replenishment over time (8). Given the high-intensity work environment and irregular schedules, nurses are particularly vulnerable to entering a cycle of resource depletion, in which fatigue is continuously reinforced and may develop into chronic occupational strain (8, 13). Therefore, understanding occupational fatigue from the perspective of resource imbalance provides a solid theoretical foundation. From the perspective of the Job Demands–Resources (JD–R) model, prolonged working hours, high workload, shift work, and work–family conflict (WFC) may be regarded as job demands that are associated with fatigue, whereas sufficient recovery opportunities and organizational support may function as important resources in reducing fatigue-related burden among clinical nurses (14). Previous evidence has also demonstrated a close association between burnout and fatigue among healthcare workers, suggesting that occupational fatigue represents an important psychosocial occupational health issue in nursing populations (15). Recent studies further suggest that demographic characteristics, including gender and age, may influence the relationship between occupational fatigue and job performance among healthcare workers (16), highlighting the potential heterogeneity of occupational fatigue experiences among nurses.

Work–family conflict (WFC) refers to a form of inter-role conflict in which the demands of work and family domains are incompatible in terms of time, strain, or behavior, making it difficult for individuals to fulfill both roles effectively (17). Due to shift work, irregular schedules, and high emotional labor demands, nurses are especially prone to experiencing bidirectional interference between work and family roles, which exacerbates role strain and psychological stress (18). Previous studies have demonstrated that Chinese nurses generally report high levels of WFC, which are closely associated with increased job stress, poorer mental health, and stronger turnover intention, making WFC an important psychosocial factor affecting nurses' occupational health (19, 20).

From a resource-based perspective, when individuals face competing demands from multiple roles under limited resource conditions, resource competition intensifies and accelerates resource depletion. When resource loss persists and recovery remains insufficient, fatigue is likely to accumulate (8). Empirical evidence has confirmed a significant positive association between WFC and occupational fatigue (21). However, most existing studies have adopted variable-centered approaches and have paid limited attention to individual heterogeneity in occupational fatigue among nurses.

To address this gap, the present study adopts a person-centered approach using latent profile analysis (LPA) to identify distinct fatigue profiles among clinical nurses. Specifically, it aims to classify occupational fatigue into latent profiles and to examine the role of work–family conflict across these profiles. In the present study, occupational fatigue was operationalized as multidimensional fatigue symptoms experienced by clinical nurses within an occupational context. This approach is expected to advance understanding of the heterogeneity of occupational fatigue and to inform targeted intervention strategies.

## 2 Methods

### 2.1 Study design and participants

This study employed a multicenter cross-sectional design. Data were collected between August 1 and September 30, 2025, in Sichuan Province, China. A convenience sampling strategy was used to recruit clinical nurses from six tertiary hospitals across different regions to increase sample diversity.

Inclusion criteria:

➀ Clinical nurses with at least 2 years of clinical nursing experience; ➁ Nurses who voluntarily agreed to participate in the online survey.

Exclusion criteria:

➀ Nurses undergoing advanced training; Nurses re-employed after retirement; ➁ Nurses who were not actively working during the survey period due to leave or other reasons.

Participation was voluntary, and completion of the anonymous questionnaire was considered as implied consent.

### 2.2 Sample size

The sample size was determined based on methodological recommendations for latent profile analysis (LPA). Previous methodological studies have suggested that a sample size of at least 300 participants is generally adequate for obtaining stable latent profile solutions and reliable parameter estimation in models using multiple continuous indicators (22). In the present study, four fatigue dimension scores derived from the Fatigue Assessment Instrument (FAI) were used as profile indicators. A total of 332 clinical nurses were included, which exceeded the recommended minimum sample size for latent profile analysis and was considered sufficient to support model stability and classification accuracy.

### 2.3 Measures

#### 2.3.1 Sociodemographic and work-related characteristics

Sociodemographic and work-related data were collected, including gender, age, marital status, education level, professional title, years of clinical experience, daily working hours, and weekly working days.

#### 2.3.2 Occupational fatigue

Multidimensional fatigue symptoms among clinical nurses were assessed using the Fatigue Assessment Instrument (FAI), originally developed by Schwartz et al. (23) and subsequently adapted into Chinese by Wang et al. (24). The instrument consists of 29 items covering four dimensions: fatigue severity, situation specificity, consequences of fatigue, and response to rest and sleep.

Although the FAI was not originally developed as a nursing-specific occupational fatigue instrument, its multidimensional structure captures fatigue severity, functional impairment, situational fatigue characteristics, and recovery-related responses, which are conceptually consistent with work-related fatigue experiences commonly observed among clinical nurses exposed to sustained occupational demands, shift work, and insufficient recovery opportunities.

Each item is rated on a 7-point Likert scale ranging from 1 (strongly disagree) to 7 (strongly agree). Dimension scores are calculated as the mean of the corresponding items, and higher scores indicate greater levels of multidimensional fatigue symptoms within an occupational nursing context.

Previous studies have demonstrated good reliability of the instrument, with Cronbach's α ranging from 0.768 to 0.916 (25). Previous studies have reported acceptable psychometric properties of the Chinese version of the FAI in healthcare-related populations. In the present study, the Cronbach's α coefficient was 0.848. The Kaiser–Meyer–Olkin (KMO) value was 0.781, and Bartlett's test of sphericity was significant (χ^2^ = 4,256.929, *P* < 0.001). Confirmatory factor analysis demonstrated acceptable residual fit indices, although several global fit indices did not fully reach recommended standards.

#### 2.3.3 Work–family conflict

Work–family conflict was measured using the Work–Family Conflict Scale (WFC), developed by Carlson et al. (26) and translated and validated in Chinese by Bai et al. (27). The scale comprises 18 items across two dimensions: work-to-family conflict and family-to-work conflict.

Each item is rated on a 5-point Likert scale ranging from 1 (strongly disagree) to 5 (strongly agree), with higher scores indicating greater levels of work–family conflict.

Previous studies have demonstrated good psychometric properties of the scale among Chinese nurses, with a Cronbach's α of 0.868 (25). In the present study, the Cronbach's α coefficient was 0.860. The KMO value was 0.860, and Bartlett's test of sphericity was significant (χ^2^ = 2,585.143, *P* < 0.001). Confirmatory factor analysis demonstrated acceptable residual fit indices, although several global fit indices remained below recommended standards.

### 2.4 Data collection

Data were collected using an online questionnaire. Prior to data collection, the research team contacted nurse managers at each participating hospital to explain the study objectives and procedures. With their assistance, the survey link was distributed to eligible nurses through WeChat.

Participants accessed and completed the questionnaire anonymously on a voluntary basis. To ensure data quality, all items were set as mandatory, and each account or IP address was restricted to a single submission. A minimum completion time of 10 min was required to reduce invalid responses. After data collection, the research team screened the responses and excluded questionnaires with logical inconsistencies or patterned answering.

### 2.5 Statistical analysis

Data analysis was performed using SPSS version 26.0 and Mplus version 8.3. Continuous variables were expressed as mean ± standard deviation, and categorical variables were presented as frequencies and percentages. Group differences were analyzed using one-way analysis of variance or chi-square tests, as appropriate. Harman's single-factor test was conducted to preliminarily assess common method bias. The first unrotated factor explained less than 40% of the total variance. However, this test provides only preliminary evidence regarding common method variance and does not eliminate the possibility of common method bias because all variables were measured using self-report questionnaires at the same time point.

Latent profile analysis (LPA) was conducted using the four dimension scores of occupational fatigue as indicator variables to identify distinct fatigue profiles among clinical nurses. The four dimension scores were used as indicators to improve model interpretability and reduce model complexity relative to item-level modeling. The use of dimension-level indicators rather than item-level indicators represented a methodological compromise intended to improve model stability and interpretability given the available sample size. Model fit was evaluated using the Akaike Information Criterion (AIC), Bayesian Information Criterion (BIC), and adjusted BIC (aBIC), with lower values indicating better fit. Entropy was used to assess classification accuracy, with values closer to 1 indicating higher precision.

The Lo–Mendell–Rubin likelihood ratio test and the bootstrap likelihood ratio test were used to compare models with different numbers of profiles. The optimal model was determined by comprehensively considering statistical fit indices (AIC, BIC, aBIC, entropy, LMRT, and BLRT), profile interpretability, classification quality, minimum class size, and substantive meaningfulness. After identifying the latent profiles, univariate analyses were conducted to examine differences in sociodemographic characteristics and work–family conflict across profiles. Multinomial logistic regression analysis was then performed with latent profile membership as the dependent variable to identify factors associated with different fatigue profiles. The low-fatigue profile was used as the reference category. Categorical variables were entered using dummy coding. Variance inflation factor (VIF) values were examined before regression analysis, with values ranging from 1.12 to 2.08, indicating no serious multicollinearity among the independent variables. Continuous variables were entered into the multinomial logistic regression model using raw total scores without standardization.

All statistical tests were two-tailed, and a *P*-value < 0.05 was considered statistically significant.

## 3 Results

### 3.1 Participant characteristics

A total of 343 questionnaires were collected, of which 332 were valid, yielding an effective response rate of 97.08%. The results of the univariate analysis showed that significant differences were observed among the latent fatigue groups in gender, marital status, years of clinical experience, daily working hours, and weekly working days (*P* < 0.05). No significant differences were found in age, education level, or professional title (*P* > 0.05). Detailed results are presented in Table 1.

**Table 1:** General characteristics of nurses and univariate analysis of different occupational fatigue profiles [*n* = 332, *n* (%)].

Variable	Category	*N* (%)	Low-fatigue group (*n* = 27)	Moderate-fatigue group (*n* = 103)	High-fatigue group (*n* = 202)	χ^2^/*F*	*P*
Gender	Male	88 (26.5)	15 (55.6)	24 (23.3)	49 (24.3)	12.764	0.002
	Female	244 (73.5)	12 (44.4)	79 (76.7)	153 (75.7)		
Age	37.29 ± 8.163		38.89 ± 6.824	36.67 ± 8.751	37.39 ± 8.163	0.828	0.438
Marital status	Unmarried	36 (10.8)	3 (11.1)	13 (12.6)	20 (9.9)	11.236	0.024
	Married	284 (85.5)	20 (74.1)	87 (84.5)	177 (87.6)		
	Divorced	12 (3.6)	4 (14.8)	3 (2.9)	5 (2.5)		
Education level	College diploma	27 (8.1)	1 (3.7)	8 (7.8)	18 (8.9)	1.146	0.887
	Bachelor's degree	273(82.2)	24 (88.9)	85 (82.5)	164 (81.2)		
	Master's degree or above	32 (9.6)	2 (7.4)	10 (9.7)	20 (9.9)		
Professional title	Junior	218 (65.7)	17 (63.0)	73 (70.9)	128 (63.4)	5.494	0.240
	Intermediate	88 (26.5)	9 (33.3)	26 (25.2)	53 (26.2)		
	Senior	26 (7.8)	1 (3.7)	4 (3.9)	21 (10.4)		
Years of clinical experience	≤ 3 years	235 (70.8)	21 (77.8)	70 (68.0)	144 (71.3)	14.232	0.027
	3–5 years	34 (10.2)	1 (3.7)	5 (4.9)	28 (13.9)		
	5–10 years	42 (12.7)	3 (11.1)	21 (20.4)	18 (8.9)		
	≥10 years	21 (6.3)	2 (7.4)	7 (6.8)	12 (5.9)		
Daily working hours	≤ 8 h	45 (13.6)	10 (37.0)	9 (8.7)	26 (12.9)	26.321	< 0.001
	8–9 h	64 (19.3)	8 (29.6)	19 (18.4)	37 (18.3)		
	9–10 h	66 (19.9)	4 (14.8)	22 (21.4)	40 (19.8)		
	10–12 h	89 (26.8)	3 (11.1)	23 (22.3)	63 (31.2)		
	≥12 h	68 (20.5)	2 (7.4)	30 (29.1)	36 (17.8)		
Weekly working days	≤ 5 d	54 (16.3)	11 (40.7)	12 (11.7)	31 (15.3)	23.160	< 0.001
	5–6 d	130 (39.2)	14 (51.9)	36 (35.0)	80 (39.6)		
	6–7 d	148 (44.6)	2 (7.4)	55 (53.4)	91 (45.0)		

### 3.2 Levels of occupational fatigue and work–family conflict

The mean total score of occupational fatigue was 22.07 ± 2.59. The scores for the four dimensions were as follows: fatigue severity (5.14 ± 1.04), situation specificity (5.21 ± 0.98), consequences of fatigue (5.42 ± 1.28), and response to rest and sleep (6.30 ± 0.96).

The mean total score of work–family conflict was 57.47 ± 9.66. The scores for work-to-family conflict and family-to-work conflict were 31.04 ± 6.17 and 26.43 ± 5.87, respectively.

### 3.3 Latent profile analysis of occupational fatigue

Latent profile analysis was conducted using the four dimensions of occupational fatigue as indicator variables. Models with one to five profiles were fitted, and the model fit indices are presented in Table 2.

**Table 2:** Model fit indices for latent profiles of occupational fatigue among clinical nurses.

Class	AIC	BIC	aBIC	***P***		Entropy	Number of classes	Class probability (%)
				LMRT	BLRT			
1	3,936.537	3,966.978	3,941.601	–	–	–	–	–
2	3,803.801	3,853.268	3,812.031	< 0.001	< 0.001	0.925	47/285	14.157/85.843
3	3,710.833	3,779.325	3,722.228	0.0219	0.0243	0.967	27/103/202	8.133/31.024/60.843
4	3,602.146	3,689.664	3,616.707	0.3037	0.3119	0.949	26/202/39/65	7.831/60.843/11.747/19.578
5	3,568.149	3,674.693	3,585.876	0.0869	0.0946	0.910	26/65/35/39/167	7.831/19.578/10.542/11.747/5.0301

As the number of profiles increased, the values of AIC, BIC, and aBIC decreased. The Lo–Mendell–Rubin test and bootstrap likelihood ratio test were significant for the three-profile model (*P* < 0.05) but became non-significant with additional profiles, indicating that adding more profiles did not substantially improve model fit.

The three-profile model demonstrated relatively high classification quality (Entropy = 0.967), acceptable subgroup proportions (>5%), and clinically interpretable profile characteristics. Compared with models containing additional profiles, the three-profile solution provided a more parsimonious and substantively interpretable classification structure. Therefore, the three-profile model was selected as the optimal solution.

To further evaluate classification precision, additional classification quality indices were examined for the selected three-profile solution. As shown in Table 3, the average posterior probabilities and classification probability matrix indicated satisfactory profile assignment precision and low cross-profile misclassification probabilities. In addition, the 95% confidence intervals for class proportions suggested acceptable estimation precision for the identified profiles.

**Table 3:** Classification quality indices and posterior probability estimates for the three-profile latent profile analysis model.

Groups	*n* (%)	95% *CI*	Average posterior probability (AvePP)	Classification probability matrix
Low-fatigue group	27 (8.13%)	5.64%−11.56%	1.000	0.998/0.002/0.000
Moderate-fatigue group	103 (31.02%)	26.22%−36.23%	0.986	0.000/0.988/0.012
High-fatigue group	202 (60.84%)	55.48%−65.96%	0.994	0.000/0.007/0.993

**Figure 1:**
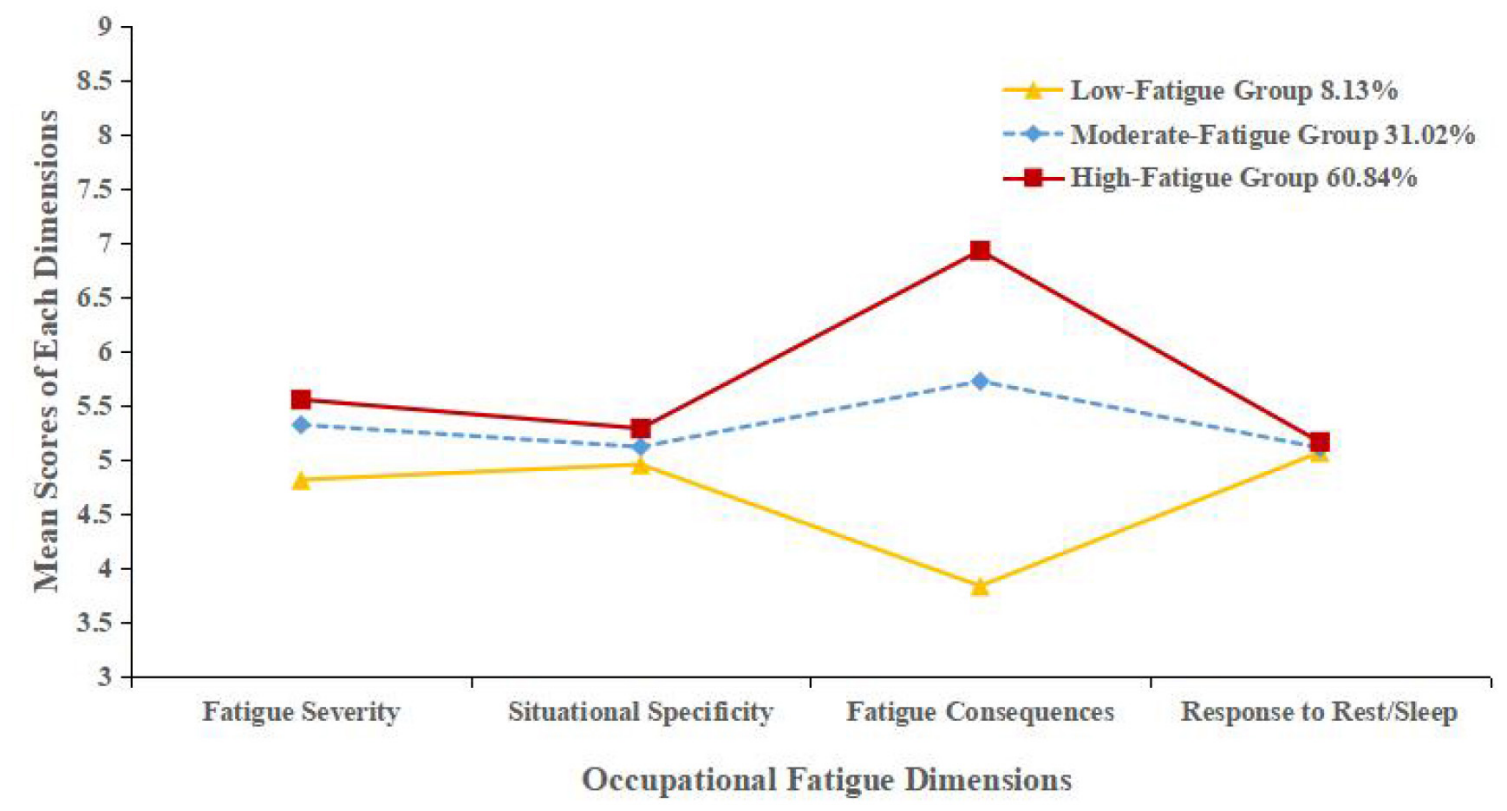
Distribution of indicator scores across latent profiles of occupational fatigue among clinical nurses.

Based on the distribution of scores across the four dimensions (Figure 1), the identified profiles were classified as low-fatigue (8.13%), moderate-fatigue (31.02%), and high-fatigue (60.84%) groups according to their overall fatigue burden and functional impairment levels.

### 3.4 Differences in work–family conflict across fatigue profiles

Significant differences in work–family conflict were observed across the three fatigue profiles (*P* < 0.001; Table 4). *Post hoc* comparisons showed a progressive increase in work–family conflict from the low-fatigue group to the high-fatigue group (*P* < 0.05).

**Table 4:** Differences in work–family conflict scores among occupational fatigue profiles in clinical nurses (mean ± *SD*).

Variable	Total (mean ±*SD*)	Low-fatigue group	Moderate-fatigue group	High-fatigue group	*F*	*P*	*Post hoc* comparison
Work–family conflict	31.04 ± 6.168	22.19 ± 3.352	30.85 ± 5.032	32.32 ± 6.013	39.734	< 0.001	C1 < C2 < C3
Family–work conflict	26.43 ± 5.866	22.33 ± 4.899	25.37 ± 6.488	27.52 ± 5.309	12.576	< 0.001	C1 < C2 < C3
Total work–family conflict score	57.47 ± 9.659	44.52 ± 6.560	56.22 ± 7.786	59.84 ± 9.399	38.210	< 0.001	C1 < C2 < C3

C1 = Low-fatigue Group; C2 = Moderate-fatigue Group; C3 = High-fatigue Group. In multinomial logistic regression analyses, the low-fatigue group (C1) was used as the reference category. OR, odds ratio; CI, confidence interval.

### 3.5 Multinomial logistic regression analysis of fatigue profiles

Multinomial logistic regression analysis was conducted with latent profile membership as the dependent variable, using the low-fatigue group as the reference. Variables that showed statistical significance in the univariate analysis were included in the model, and their coding is presented in Table 5. The overall model was statistically significant (χ^2^ = 158.772, *P* < 0.001).

**Table 5:** Coding scheme for independent variables.

Variable	Mode of assignment
Gender	1 = male, 2 = female
Marital status	1 = unmarried, 2 = married, 3 = divorced
Years of clinical experience	1 = ≤ 3 years, 2 = 3–5 years, 3 = 5–10 years,4 = ≥10 years
Daily working hours	1 = ≤ 8 h, 2 = 8–9 h, 3 = 9–10 h, 4 = 10–12 h, 5 = ≥12 h
Weekly working days	1 = ≤ 5 d, 2 = 5–6 d, 3 = 6–7 d
Total work–family conflict score	Continuous variables

Work–family conflict was significantly associated with greater likelihood of belonging to more severe occupational fatigue profiles. In contrast, male nurses were less likely to be classified into the moderate- and high-fatigue groups than female nurses.

Regarding work-related factors, shorter daily working hours and fewer weekly working days were associated with lower likelihood of belonging to the moderate- and high-fatigue groups. In addition, longer daily working hours were associated with greater likelihood of membership in the high-fatigue group. Detailed results are presented in Table 6.

**Table 6:** Multinomial logistic regression analysis of latent profiles of occupational fatigue among clinical nurses (*n* = 332).

Variable	Reference	β	*SE*	Wald χ^2^	*P*	*OR*	95% *CI*
C2 vs. C1							
Constant	–	−12.646	3.891	10.563	0.001	–	–
Gender	Female	−2.003	0.748	7.172	0.007	0.135	0.031–0.584
Daily working hours							
≤ 8 h	≥12 h	−3.435	1.262	7.410	0.006	0.032	0.003–0.382
8–9 h		−2.878	1.217	5.590	0.018	0.056	0.005–0.611
Weekly working days ≤ 5 d	6–7 d	−3.058	1.207	6.419	0.011	0.047	0.004–0.500
Total work–family conflict score	–	0.338	0.078	18.718	< 0.001	1.403	1.203–1.635
C3 vs. C1							
Constant	–	−16.216	3.912	17.181	< 0.001	–	–
Gender	Female	−2.018	0.741	7.426	0.006	0.133	0.031–0.567
Daily working hours							
≤ 8 h	≥12 h	−2.470	1.246	3.933	0.047	0.085	0.007–0.971
8–9 h		−2.531	1.219	4.313	0.038	0.080	0.007–0.867
Weekly working days ≤ 5 d	6–7 d	−2.396	1.200	3.983	0.046	0.091	0.009–0.958
Total work–family conflict score		0.394	0.078	25.296	< 0.001	1.483	1.272–1.730
C3 vs. C2							
Constant	–	−3.570	1.335	7.150	0.007	–	–
Daily working hours 10–12 h	≥12 h	0.837	0.369	5.155	0.023	2.310	1.121–4.757
Total work–family conflict score	–	0.056	0.016	11.690	< 0.001	1.057	1.024–1.092

C1 = Low-fatigue group; C2 = moderate-fatigue Group; C3 = high-fatigue group; OR, odds ratio; CI, confidence interval.

## 4 Discussion

### 4.1 High levels of occupational fatigue among clinical nurses

The findings of this study indicate that clinical nurses experience relatively high levels of occupational fatigue, with particularly elevated scores observed in fatigue consequences and recovery-related dimensions. This suggests that fatigue has already exerted a measurable impact on functional status. These findings are consistent with previous studies reporting a high prevalence of fatigue among nurses (15, 28).

Several occupational characteristics may underlie this pattern. Clinical nurses are routinely exposed to high-intensity workloads, shift work, and substantial emotional demands, all of which may increase physical and psychological strain while limiting opportunities for recovery (15, 29). In addition, insufficient staffing further exacerbates the imbalance between workload and recovery, thereby contributing to higher fatigue burden (30). At a broader level, these findings may also reflect structural challenges in healthcare resource allocation, which may intensify sustained occupational strain and heavier fatigue burden among clinical nurses (31). The multidimensional structure of the FAI may provide a broader perspective for understanding fatigue-related experiences among clinical nurses within occupational settings.

### 4.2 Heterogeneity of occupational fatigue profiles

This study identified three occupational fatigue profile patterns among clinical nurses, namely the low-fatigue group, moderate-fatigue group, and high-fatigue group, indicating substantial heterogeneity in occupational fatigue levels. Notably, the proportion of nurses classified into the high-fatigue profile reached 60.84%, which was higher than that reported in some previous studies (8, 29, 32). This finding suggests that occupational fatigue in this sample may partly reflect severity gradients across fatigue dimensions rather than completely discrete latent categories. Therefore, the identified profiles should be interpreted as empirically derived profile patterns reflecting different levels and configurations of fatigue rather than definitive clinical subtypes (33, 34).

The low-fatigue group (8.13%) was primarily composed of nurses with shorter daily working hours ( ≤ 8 h) and fewer weekly working days ( ≤ 5 days), suggesting a relatively lower workload level. Under relatively lower work intensity and frequency, physical and cognitive resource demands may be comparatively reduced, while sufficient recovery opportunities may help maintain lower levels of occupational fatigue (35, 36).

The moderate-fatigue group (31.02%) was mainly characterized by nurses with ≤ 3 years of clinical experience, working 6–7 days per week and ≥9 h per day, with a higher proportion of females. Although these nurses were in the early stage of their careers, they were already exposed to relatively high work intensity and frequency. Higher levels of workload and limited recovery opportunities may be associated with relatively higher fatigue burden in this group (8, 37).

The high-fatigue group (60.84%) was predominantly composed of nurses working ≥10 h per day and 6–7 days per week, with a higher proportion of married female nurses. Prolonged working hours, frequent work schedules, and family-related responsibilities may collectively contribute to higher resource demands and fewer recovery opportunities, which may help explain the relatively higher occupational fatigue levels observed in this group (12, 38, 39).

Overall, the identified occupational fatigue profiles may reflect different levels and configurations of fatigue burden among clinical nurses. These findings further support the heterogeneous nature of occupational fatigue and may provide additional insight into fatigue-related workload and psychosocial characteristics in nursing populations. However, the identified profiles may partly reflect severity gradients across the observed fatigue burden rather than fully discrete latent categories.

### 4.3 The role of work–family conflict in occupational fatigue

This study found significant differences in work–family conflict across different occupational fatigue profiles, with levels increasing progressively from the low-fatigue group to the high-fatigue group. Multinomial logistic regression analysis further confirmed that work–family conflict was significantly associated with both moderate and high levels of occupational fatigue, highlighting its close relationship with occupational fatigue profiles.

According to the Conservation of Resources (COR) theory, individuals possess limited time, energy, and emotional capacity. When both work and family roles simultaneously demand these capacities, competition may arise, increasing psychological strain and reducing recovery opportunities (8, 40). In this context, work–family conflict may represent an important work-related stress factor linked to occupational fatigue (40).

Work demands may spill over into the family domain and reduce opportunities for rest and recovery, while family responsibilities may further increase psychosocial strain (41, 42). This dual pressure may reduce opportunities for recovery and increase fatigue burden among clinical nurses.

Across fatigue profiles, higher work–family conflict scores were observed among nurses with more severe fatigue profiles. This pattern suggests that work–family conflict appears closely linked to more severe occupational fatigue profiles, particularly when combined with high workload and limited recovery opportunities.

Overall, work–family conflict was significantly associated with occupational fatigue profiles and may help explain heterogeneity in fatigue burden from a psychosocial work–family interface perspective.

### 4.4 The impact of workload on occupational fatigue

The results of this study showed that daily working hours and weekly working days were significantly associated with latent profiles of occupational fatigue. As working hours increased and work frequency became higher, the likelihood of nurses belonging to the high-fatigue group also increased, whereas lower workload levels were more commonly observed in the low-fatigue profile. These findings are consistent with previous studies, indicating that workload is an important external factor influencing occupational fatigue (12, 43).

From the perspective of the Job Demands–Resources (JD–R) model, prolonged working hours and frequent work schedules may represent excessive job demands that increase fatigue burden when recovery opportunities and organizational resources are insufficient (14). Prolonged working hours and high-frequency work may increase physical and cognitive demands while restricting opportunities for sleep and psychological recovery. These work patterns may therefore be associated with more severe fatigue profiles among clinical nurses (8, 44).

Consistent with the latent profile results, the high-fatigue group was predominantly concentrated among nurses with higher workload levels, whereas the low-fatigue group was more commonly observed among those with lower workload levels. These findings further support the close relationship between workload-related demands and more severe occupational fatigue profiles.

### 4.5 The influence of individual and occupational characteristics

This study found that individual and occupational characteristics, including gender, marital status, and years of clinical experience, differed across occupational fatigue profiles, suggesting that these factors are linked to differences in occupational fatigue profiles.

Specifically, female nurses were more likely to belong to the moderate- and high-fatigue groups, which may be related to the greater emotional labor demands and additional family responsibilities they often undertake, both of which increase additional psychosocial burden (15).

Years of clinical experience showed a stage-specific association with occupational fatigue. The moderate-fatigue group was mainly composed of nurses with ≤ 3 years of clinical experience, suggesting that nurses in the early stage of their careers are more likely to experience fatigue under conditions of limited clinical experience and limited coping capacity (37, 45). As clinical experience increases, individuals may gradually develop more effective coping strategies and coping abilities, which may be related to lower fatigue burden (8, 46).

Overall, occupational fatigue is influenced not only by workload but also by individual characteristics and their capacity for resource regulation, further reflecting the heterogeneous nature of occupational fatigue.

Overall, the findings of the present study may be understood within multiple occupational stress frameworks. COR theory emphasizes the role of limited personal resources and recovery opportunities in fatigue patterns, whereas the JD–R model highlights the imbalance between excessive job demands and insufficient occupational resources (14). In addition, effort–reward imbalance theory may provide a useful interpretative perspective for understanding associations between perceived occupational imbalance and fatigue-related experiences among clinical nurses. Together, these frameworks provide complementary theoretical perspectives for contextualizing the heterogeneous nature of occupational fatigue among clinical nurses.

## 5 Conclusions and implications

### 5.1 Conclusions

This study identified three occupational fatigue profile groups identified by latent profile analysis among clinical nurses, namely the low-fatigue group, moderate-fatigue group, and high-fatigue group, demonstrating substantial heterogeneity in fatigue levels. Significant differences were observed across these profiles in terms of workload, work–family conflict, and individual and occupational characteristics, indicating that occupational fatigue arises from the combined effects of multiple factors.

Further analysis revealed that workload, reflected by daily working hours and weekly working days, is an important external factor contributing to occupational fatigue. Excessive workload may be associated with increased resource demands and fewer recovery opportunities, which may help explain the more severe occupational fatigue profiles observed among clinical nurses. In addition, work–family conflict may be associated with occupational fatigue through increased competition for personal resources and limited recovery opportunities. Individual and occupational characteristics, including gender, marital status, and years of clinical experience, also influence fatigue levels to varying degrees, further reflecting the heterogeneous nature of occupational fatigue.

### 5.2 Implications

Based on the findings of this study, several targeted strategies are recommended:

(1) Optimizing scheduling and workload management
  Given the association between workload and occupational fatigue, nursing managers should aim to reduce prolonged working hours and consecutive working days when arranging schedules. Appropriate shift intervals and flexible scheduling strategies may help minimize sustained high workload exposure and reduce continuous resource depletion.
(2) Reducing work–family conflict
  For nurses experiencing high levels of work–family conflict, scheduling arrangements may take into account family responsibilities where possible. Establishing flexible shift adjustment mechanisms and maintaining regular communication may help alleviate role conflict and reduce the negative spillover of work demands into the family domain.
(3) Implementing profile-based fatigue management
  Based on the identified fatigue profiles, regular fatigue assessment may be conducted, and stratified management strategies may be implemented. For nurses in the high-fatigue group, reducing consecutive night shifts and prolonged working hours may be necessary. For those in the moderate-fatigue group, controlling workload intensity and frequency may help prevent further fatigue accumulation. For the low-fatigue group, maintaining relatively stable work arrangements may help sustain their current state.
(4) Providing targeted support for early-career and female nurses
  Nurses with shorter clinical experience may benefit from enhanced training and mentorship to reduce the additional burden associated with limited experience. For female nurses, particularly those with family responsibilities, appropriate consideration in scheduling and role allocation may help reduce resource strain arising from multiple role demands.

## 6 Limitations and future directions

This study has several limitations. First, the cross-sectional design precludes any inference regarding causal or temporal relationships among variables. Therefore, the associations observed between work–family conflict and occupational fatigue should be interpreted cautiously, and no conclusions can be drawn regarding developmental variation or directional mechanisms. Future studies are encouraged to adopt longitudinal designs to further examine the dynamic relationships and potential profile differences between occupational fatigue profiles over time.

Second, the sample was recruited using convenience sampling from six tertiary hospitals in Sichuan Province, which may introduce potential selection bias and limit the generalizability of the findings across different healthcare settings. In particular, nurses experiencing higher levels of occupational fatigue or work–family conflict may have been more motivated to participate in the survey. Future studies are encouraged to include more diverse healthcare settings and adopt broader sampling strategies to improve external validity.

Third, the data were primarily based on self-reported questionnaires, which may be subject to information bias, social desirability effects, and common method bias associated with mono-method assessment. Future studies are encouraged to incorporate multi-source data, longitudinal designs, or objective indicators to further reduce potential common method bias and strengthen the robustness of the findings.

In addition, although latent profile analysis identified different occupational fatigue profile patterns, the stability of the identified profiles requires further validation in future studies using larger samples and alternative modeling strategies. Sensitivity analyses using alternative model specifications were not conducted in the present study, and the identified profile structure therefore requires further validation in future research. In addition, although confirmatory factor analyses were conducted for the study instruments, several model fit indices did not fully meet recommended standards, and the dimensional structures of the scales should therefore be interpreted with appropriate caution in this sample.

Future research is also encouraged to explore occupational fatigue from a dynamic perspective, with particular attention to the profile differences mechanisms between different fatigue profiles. In addition, intervention-based studies may be conducted to evaluate the effectiveness of strategies targeting workload and work–family conflict in reducing occupational fatigue, thereby providing stronger evidence to inform clinical practice.

## Data Availability

The original contributions presented in the study are included in the article/supplementary material, further inquiries can be directed to the corresponding author.

## References

[1] Coomber B Barriball KL. Impact of job satisfaction components on intent to leave and turnover for hospital-based nurses: a review of the research literature. Int J Nurs Stud. (2007) 44:297–314. doi: 10.1016/j.ijnurstu.2006.02.004 16631760

[2] Griffiths P Ball J Drennan J Dall'Ora C Jones J Maruotti A . Nurse staffing and patient outcomes: strengths and limitations of the evidence to inform policy and practice. A review and discussion paper based on evidence reviewed for the National Institute for Health and Care Excellence Safe Staffing guideline development. Int J Nurs Stud. (2016) 63:213–25. doi: 10.1016/j.ijnurstu.2016.03.012 27130150

[3] Tamata AT Mohammadnezhad M. A systematic review study on the factors affecting shortage of nursing workforce in the hospitals. Nurs Open. (2023) 10:1247–57. doi: 10.1002/nop2.1434 36303066 PMC9912424

[4] Mohamed Z Al-Hmaimat N. The effectiveness of nurse residency programs on new graduate nurses' retention: systematic review. Heliyon. (2024) 10:e26272. doi: 10.1016/j.heliyon.2024.e26272 38434316 PMC10907523

[5] Cai S Lin H Hu X Cai YX Chen K Cai WZ. High fatigue and its associations with health and work related factors among female medical personnel at 54 hospitals in Zhuhai, China. Psychol Health Med. (2018) 23:304–16. doi: 10.1080/13548506.2017.1361038 28778141

[6] Department Department of Planning Development and Information Technology National National Health Commission of the People's Republic of China. Statistical bulletin of China's health development in 2023. Chin J Viral Dis. (2024) 416–24.

[7] Wang LY Hu ZY Chen HX Zhou CF Tang ML Hu XY. Differences in regional distribution and inequality in health workforce allocation in hospitals and primary health centers in China: a longitudinal study. Int J Nurs Stud. (2024) 157:104816. doi: 10.1016/j.ijnurstu.2024.104816 38824719

[8] Hobfoll SE. Conservation of resources. A new attempt at conceptualizing stress. Am Psychol. (1989) 44:513–24. doi: 10.1037/0003-066X.44.3.513 2648906

[9] Zeng Z Zhou S Xie G He Y Ling J. The relationship between sleep quality and occupational fatigue in endoscopy nurses: mediating role of positive coping style. Front Public Health. (2024) 12:1437659. doi: 10.3389/fpubh.2024.1437659 39026595 PMC11254638

[10] Sagherian K Clinton ME Abu-Saad Huijer H Geiger-Brown J. Fatigue, work schedules, and perceived performance in bedside care nurses. Workplace Health Saf . (2017) 65:304–12. doi: 10.1177/2165079916665398 27872407

[11] Crincoli S de Cordova P Thomas-Hawkins C Flynn L Zha P Sagherian K. The effects of organizational characteristics, individual nurse characteristics, and occupational fatigue on missed care at night. Nurs Res. (2024) 73:101–8. doi: 10.1097/NNR.0000000000000696 37862123

[12] Dall'Ora C Ball J Recio-Saucedo A Griffiths P. Characteristics of shift work and their impact on employee performance and wellbeing: a literature review. Int J Nurs Stud. (2016) 57:12–27. doi: 10.1016/j.ijnurstu.2016.01.007 27045561

[13] Drach-Zahavy A Marzuq N. The weekend matters: exploring when and how nurses best recover from work stress. J Adv Nurs. (2013) 69:578–89. doi: 10.1111/j.1365-2648.2012.06033.x 22606992

[14] Demerouti E Bakker AB Nachreiner F Schaufeli WB. The job demands-resources model of burnout. J Appl Psychol. (2001) 86:499–512. doi: 10.1037/0021-9010.86.3.499 11419809

[15] Woo T Ho R Tang A Tam W. Global prevalence of burnout symptoms among nurses: a systematic review and meta-analysis. J Psychiatr Res. (2020) 123:9–20. doi: 10.1016/j.jpsychires.2019.12.015 32007680

[16] Uysal D Potas N. Controlled role of gender and age between work fatigue and job performance in healthcare managers. J Manag Decis. (2026). doi: 10.1108/MD-02-2025-0430

[17] Haji Matarsat HM Rahman HA Abdul-Mumin K. Work-family conflict, health status and job satisfaction among nurses. Br J Nurs. (2021) 30:54–8. doi: 10.12968/bjon.2021.30.1.54 33433277

[18] Aldhafeeri NA Abou Hashish EA Abo Shereda HM. The effect of work-family conflict on staff nurses' job performance: the mediating role of emotional intelligence. BMC Nurs. (2025) 24:614. doi: 10.1186/s12912-025-03280-w 40448128 PMC12124067

[19] Yang Q Yang L Yang C Wu X Xu Z Wang X. How is work-family conflict linked to nurse-assessed patient safety among intensive care unit nurses? A serial multiple mediation analysis. Aust Crit Care. (2025) 38:101053. doi: 10.1016/j.aucc.2024.03.008 38762342

[20] Diao D Chen X Zhong L Zhang H Zhang J. Sex differences in burnout and work-family conflict among Chinese emergency nurses: a cross-sectional study. Front Public Health. (2024) 12:1492662. doi: 10.3389/fpubh.2024.1492662 39712298 PMC11659251

[21] Zeng Z He Y Liao X Song Y Zhou S. Occupational fatigue and its determinants among endoscopy nurses in China: a cross-sectional study with structural equation modeling. Front Public Health. (2026) 14:1759966. doi: 10.3389/fpubh.2026.1759966 41717625 PMC12913506

[22] Nylund-Gibson K Garber AC Carter DB Chan M Arch DAN Simon O . Ten frequently asked questions about latent transition analysis. Psychol Methods. (2023) 28:284–300. doi: 10.1037/met0000486 35834194

[23] Schwartz JE Jandorf L Krupp LB. The measurement of fatigue: a new instrument. J Psychosom Res. (1993) 37:753–62. doi: 10.1016/0022-3999(93)90104-N 8229906

[24] Wang T Zhang C Liu Y Jiao YA. Study on the characteristic of fatigue, depression, anxiety, life event and their correlation in the patients with chronic fatigue syndrome. Chin J Behav Med Brain Sci. (2000) 8–10.

[25] Yao Y Tang J Meng H Li Y Du H Li Z. The mediating effect of psychological flexibility on fatigue and depressive symptoms among nursing staff. Int J Occup Med Environ Health. (2023) 36:563–74. doi: 10.13075/ijomeh.1896.02073 37964727 PMC10691421

[26] Carlson DS Kacmar KM Williams LJ. Construction and initial validation of a multidimensional measure of work–family conflict. J Vocat Behav. (2000) 56:249–76. doi: 10.1006/jvbe.1999.1713

[27] Bai J Fang HL Sun RN. Status quo and influencing factors of work-family conflict in 552 clinical nurses. J Nurs. (2020) 27:38–41.

[28] Nemati-Vakilabad R Ebadi E Homaei A Hoseini S Mirzaei A. The relationship between perceived nursing workload and occupational fatigue in clinical nurses: the moderating role of nursing teamwork. J Clin Nurs. (2025) 34:4132–41. doi: 10.1111/jocn.17616 39654037

[29] Cao Y Dong Y Shi L Chappell K Jia Z Yan T . Occupational burnout in nurses is due to long-term work stress rather than COVID-19 pandemic event. J Adv Nurs. (2026) 82:3598–616. doi: 10.1111/jan.17085 40432559

[30] Aiken LH Clarke SP Sloane DM Sochalski J Silber JH. Hospital nurse staffing and patient mortality, nurse burnout, and job dissatisfaction. JAMA. (2002) 288:1987–93. doi: 10.1001/jama.288.16.1987 12387650

[31] Dong E Wang T Xu T Chen X Zhou Q Gao W . What are the key factors contributing to the inequity in healthcare resource allocation? Evidence from China's health panel data from 2009 to 2021. Front Public Health. (2025) 13:1586585. doi: 10.3389/fpubh.2025.1586585 40756397 PMC12313659

[32] Seidler A Thinschmidt M Deckert S Then F Hegewald J Nieuwenhuijsen K . The role of psychosocial working conditions on burnout and its core component emotional exhaustion—a systematic review. J Occup Med Toxicol. (2014) 9:10. doi: 10.1186/1745-6673-9-10 24628839 PMC4233644

[33] Padmanabhanunni A Pretorius TB. Trauma exposure, insomnia, and fatigue: a cross-sectional study of the pathways to burnout among South African First responders. Health Sci Rep. (2025) 8:e71204. doi: 10.1002/hsr2.71204 40895245 PMC12394180

[34] Portoghese I Min A Galletta M. Latent profiles of job demands and job resources and their association with work wellbeing among nurses in South Korea. Sci Rep. (2025) 15:16439. doi: 10.1038/s41598-025-01479-0 40355607 PMC12069683

[35] Sonnentag S Venz L Casper A. Advances in recovery research: what have we learned? What should be done next? J Occup Health Psychol. (2017) 22:365–80. doi: 10.1037/ocp0000079 28358572

[36] Han S Kwak S. The effect of sleep disturbance on the association between work-family conflict and burnout in nurses: a cross-sectional study from South Korea. BMC Nurs. (2022) 21:354. doi: 10.1186/s12912-022-01114-7 36510296 PMC9742643

[37] Laschinger HK Grau AL. The influence of personal dispositional factors and organizational resources on workplace violence, burnout, and health outcomes in new graduate nurses: a cross-sectional study. Int J Nurs Stud. (2012) 49:282–91. doi: 10.1016/j.ijnurstu.2011.09.004 21978860

[38] Sandoval-Reyes J Restrepo-Castro JC Duque-Oliva J. Work intensification and psychological detachment: the mediating role of job resources in health service workers. Int J Environ Res Public Health. (2021) 18:12228. doi: 10.3390/ijerph182212228 34831983 PMC8624283

[39] La Torre G Grima D Romano F Polimeni A. Perceived work ability and work-family conflict in healthcare workers: an observational study in a teaching hospital in Italy. J Occup Health. (2021) 63:e12271. doi: 10.1002/1348-9585.12271 34535041 PMC8448582

[40] Ten Brummelhuis LL Bakker AB. A resource perspective on the work-home interface: the work-home resources model. Am Psychol. (2012) 67:545–56. doi: 10.1037/a0027974 22506688

[41] Frone MR. Work-family conflict and employee psychiatric disorders: the National Comorbidity Survey. J Appl Psychol. (2000) 85:888–95. doi: 10.1037/0021-9010.85.6.888 11155895

[42] Chen IS. Extending the job demands-resources model to understand the effect of the interactions between home and work domains on work engagement. Stress Health. (2024) 40:e3362. doi: 10.1002/smi.3362 38197865

[43] Zhong L Wang L Zhang H Diao D Chen X Zou L. Effort-reward imbalance among emergency department nurses in China: construction and evaluation of a nomogram predictive model. J Nurs Manag. (2025) 2025:1412700. doi: 10.1155/jonm/1412700 40510889 PMC12162163

[44] Bouchou Y Monnier M Roche F Pélissier C Berger M. Effect of 12 weeks with a 30-min nap opportunity during the night shift of healthcare workers on early cardiovascular risk biomarkers: the NAPWORK study protocol of a randomised controlled trial. BMJ Open. (2026) 16:e110108. doi: 10.1136/bmjopen-2025-110108 41571410 PMC12829368

[45] Kaldal MH Voldbjerg SL Grønkjaer M Conroy T Feo R. Newly graduated nurses' commitment to the nursing profession and their workplace during their first year of employment: a focused ethnography. J Adv Nurs. (2024) 80:1058–71. doi: 10.1111/jan.15883 37792389

[46] Chang Y Chan HJ. Optimism and proactive coping in relation to burnout among nurses. J Nurs Manag. (2015) 23:401–8. doi: 10.1111/jonm.12148 24112222

